# Supplementary material for: An adaptive, continuous-learning framework for clinical decision-making from proteome-wide biofluid data
Source: Nat Commun. 2026 Jan 27;17:1105. doi: 10.1038/s41467-025-67968-y (PMC12852910; doi:10.1038/s41467-025-67968-y)
Supplement: Supplementary file 1 — Supplementary Information [file 41467_2025_67968_MOESM1_ESM.pdf]

# Supplementary Information

## An adaptive, continuous-learning framework for clinical decision-making from proteome-wide biofluid data

Johannes B. Müller-Reif, Vincent Albrecht, *et al.* (2025)

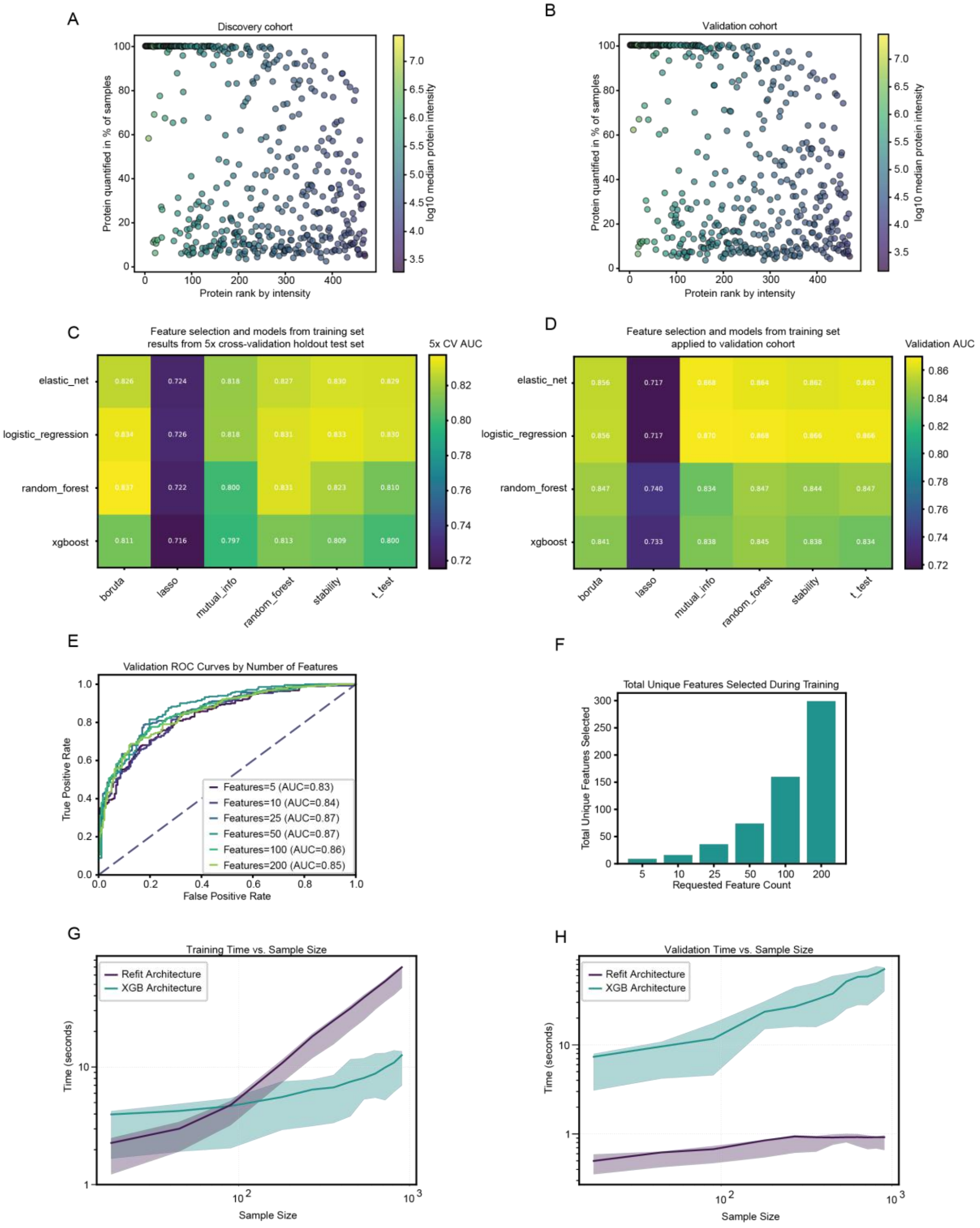

***Supplementary Fig. 1: Additional metrics to application of ADAPT-MS to plasma proteomics sepsis cohort.*** *(A) Protein rank vs data completeness plot for all proteins quantified in the sepsis discovery cohort. (B) Protein rank vs data completeness plot for all proteins quantified in the sepsis validation cohort. (C) Grid search results of the combination of various classifiers and feature selection methods in a 5x-crossvalidation manner for the discovery set (ROC AUC values). (D) Grid search results of the combination of various classifiers and feature selection methods applied to the validation set (ROC AUC values). (E) Number of features selected per iteration of 5x cross-validation in the feature selection evaluated on ADAPT-MS model performance. (F) Total number of features selected during the feature selection step by t-test in the 5x cross-validation manner. The number of unique features selected represents the relaxed feature list going into the ADAPT-MS architecture. (G-H) Algorithm run time for XGBoost architecture vs ADAPT-MS architecture dependent on number of samples included for training for the training procedure and validation procedure.*

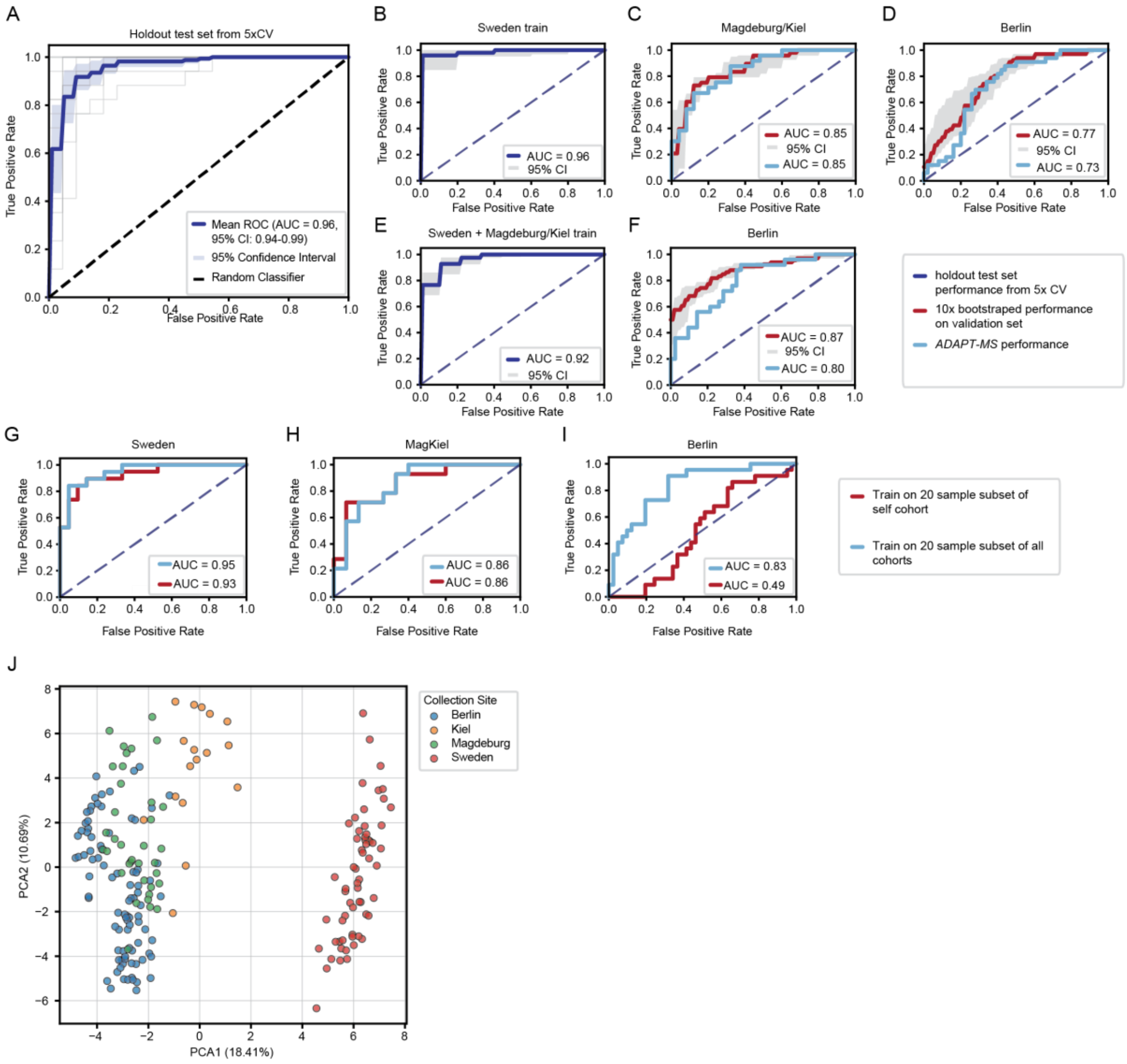


***Supplementary Fig. 2: Additional metrics for application of ADAPT-MS to the CSF AD cohort.*** *(A) ROC AUC of a random forest feature selection, XGBoost classification model on the complete AD CSF cohort in 5x cross-validation manner, reproducing and exceeding the originally published classification performance. (B) 5x cross-validation results of the Sweden training cohort set for control and ADAPT-MS architecture application on Magdeburg/Kiel (C) and Berlin (D) cohort. (E) 5x cross-validation results of the Sweden + Magdeburg/Kiel training cohort set for control and ADAPT-MS architecture application on Berlin cohort (F). (G-I) application of ADAPT-MS using either a 20-sample subset of all cohort for feature selection or just a 20-sample subset of the self-cohort for training. (J) Principal component analysis of CSF samples from the different sub-cohorts of the reused study data.*
